# Genomic features of rapid versus late relapse in triple negative breast cancer

**DOI:** 10.1186/s12885-021-08320-7

**Published:** 2021-05-18

**Authors:** Yiqing Zhang, Sarah Asad, Zachary Weber, David Tallman, William Nock, Meghan Wyse, Jerome F. Bey, Kristin L. Dean, Elizabeth J. Adams, Sinclair Stockard, Jasneet Singh, Eric P. Winer, Nancy U. Lin, Yi-Zhou Jiang, Ding Ma, Peng Wang, Leming Shi, Wei Huang, Zhi-Ming Shao, Mathew Cherian, Maryam B. Lustberg, Bhuvaneswari Ramaswamy, Sagar Sardesai, Jeffrey VanDeusen, Nicole Williams, Robert Wesolowski, Samilia Obeng-Gyasi, Gina M. Sizemore, Steven T. Sizemore, Claire Verschraegen, Daniel G. Stover

**Affiliations:** 1 Ohio State University College of Medicine, 370 W 9th Ave, Columbus, OH 43210 USA; 2 Division of Medical Oncology, Ohio State University Comprehensive Cancer Center, 460 W 10th Ave, Columbus, OH 43210 USA; 3 Department of Biomedical Informatics, The Ohio State University, Columbus, OH 43210 USA; 4 Stefanie Spielman Comprehensive Breast Center, 1145 Olentangy River Rd, Columbus, OH 43212 USA; 5 Department of Medical Oncology, Susan F. Smith Center for Women’s Cancers, Dana-Farber Cancer Institute, 450 Brookline Avenue, Boston, MA 02215 USA; 6 Department of Breast Surgery, Precision Cancer Medicine Center, Fudan University Shanghai Cancer Center, 270 Dong’an Road, Shanghai, 200032 P.R. China; 7 Shanghai Institute of Nutrition and Health, Shanghai Institutes for Biological Sciences, University of Chinese Academy of Sciences, Chinese Academy of Sciences, 320 Yueyang Road, Shanghai, 200031 P.R. China; 8 State Key Laboratory of Genetic Engineering, School of Life Sciences and Human Phenome Institute, Fudan University, 2005 Songhu Road, Shanghai, 200438 P.R. China; 9 Shanghai-MOST Key Laboratory of Health and Disease Genomics, Chinese National Human Genome Center at Shanghai (CHGC) and Shanghai Industrial Technology Institute (SITI), 250 Bibo Road, Shanghai, 201203 P.R. China; 10 Stefanie Spielman Comprehensive Breast Center, Ohio State University Comprehensive Cancer Center, Biomedical Research Tower, Room 512, Columbus, OH 43210 USA

**Keywords:** Breast Cancer, Triple-negative breast cancer, Machine learning

## Abstract

**Background:**

Triple-negative breast cancer (TNBC) is a heterogeneous disease and we have previously shown that rapid relapse of TNBC is associated with distinct sociodemographic features. We hypothesized that rapid versus late relapse in TNBC is also defined by distinct clinical and genomic features of primary tumors.

**Methods:**

Using three publicly-available datasets, we identified 453 patients diagnosed with primary TNBC with adequate follow-up to be characterized as ‘rapid relapse’ (rrTNBC; distant relapse or death ≤2 years of diagnosis), ‘late relapse’ (lrTNBC; > 2 years) or ‘no relapse’ (nrTNBC: > 5 years no relapse/death). We explored basic clinical and primary tumor multi-omic data, including whole transcriptome (*n* = 453), and whole genome copy number and mutation data for 171 cancer-related genes (*n* = 317). Association of rapid relapse with clinical and genomic features were assessed using Pearson chi-squared tests, t-tests, ANOVA, and Fisher exact tests. We evaluated logistic regression models of clinical features with subtype versus two models that integrated significant genomic features.

**Results:**

Relative to nrTNBC, both rrTNBC and lrTNBC had significantly lower immune signatures and immune signatures were highly correlated to anti-tumor CD8 T-cell, M1 macrophage, and gamma-delta T-cell CIBERSORT inferred immune subsets. Intriguingly, lrTNBCs were enriched for luminal signatures. There was no difference in tumor mutation burden or percent genome altered across groups. Logistic regression mModels that incorporate genomic features significantly outperformed standard clinical/subtype models in training (*n* = 63 patients), testing (*n* = 63) and independent validation (*n* = 34) cohorts, although performance of all models were overall modest.

**Conclusions:**

We identify clinical and genomic features associated with rapid relapse TNBC for further study of this aggressive TNBC subset.

**Supplementary Information:**

The online version contains supplementary material available at 10.1186/s12885-021-08320-7.

## Background

Triple negative breast cancer (TNBC) is an aggressive breast cancer subtype defined by lack of targetable estrogen receptor (ER), progesterone receptor (PR), and HER2 [1]. TNBC accounts for 15% of breast cancer cases, yet is responsible for 35% of breast cancer related deaths [1, 2]. Relative to hormone receptor positive breast cancers, TNBCs are more likely to develop distant rather than local recurrence and TNBCs spread more frequently to visceral sites, including lung and brain [2–4]. Understanding determinants of distant relapse is imperative as the median overall survival after diagnosis of metastatic disease was historically only 13–17 months [2, 5] and remains only 25 months even among patients with PD-L1 positive TNBC receiving chemo-immunotherapy [6].

Advances in sequencing technology have facilitated comprehensive molecular profiling of breast cancers, including subsets of TNBC [7, 8]. Two landmark analysis of primary TNBCs revealed six subtypes of TNBC with distinct expression profiles [9, 10] and an integrated copy number/transcriptome analysis identified four overlapping TNBC subsets [11]. Genomic analyses demonstrate high frequency of mutations in *TP53* (~ 75% of TNBCs) and *PIK3CA* ~ 25% [11–13] while TNBCs also reflect widespread copy number alterations [11–13]. The existing TNBC subsets/groupings provide a critical framework for understanding intrinsic genomic characteristics but are only associated with modest differences in patient survival. Among the approximately 30% of TNBCs who develop metastatic disease, a subset have an aggressive phenotype associated with rapid relapse, therapeutic resistance, and poor prognosis, while others have a relatively late relapse associated with more indolent or treatment responsive disease – yet we have a poor understanding of genomic features associated with distinct timing of relapse [1, 2, 14].

To more accurately understand the differences in patient outcome in TNBC, we sought to understand distinct clinical and genomic features among primary TNBCs categorized based on outcome: rapid (rrTNBC), late (lrTNBC) and no relapse (nrTNBC). In several large TNBC cohort studies, the median time to distant metastasis was around 2 years, ranging from 19.7 to 31.2 months, [2, 14–16] thus we define rrTNBC as relapse or death within 24 months of diagnosis. We previously demonstrated in two large cohorts (Surveillance, Epidemiology, and End Results Program/SEER and National Comprehehensive Cancer Network/NCCN) that disparities in sociodemographic features are strongly associated with rrTNBC, including insurance type, race, and surgical management [17–19]. These studies demonstrate the relevance of understanding factors contributing to rrTNBC yet are limited by lack of biologic understanding.

As an initial investigation of genomic features associated with rrTNBC, we aggregated data across multiple cohorts then utilized a train/test split and an independent validation cohort to model predictors of rapid versus late relapse.

## Methods

### Patient and tumor characteristics

Patient-specific data were obtained from The Cancer Genome Atlas (TCGA) [12], Molecular Taxonomy of Breast Cancer International Consortium (METABRIC) [20, 21], our published meta-analysis (“neoadjuvant dataset” as described previously) [7], and the Fudan TNBC cohort [22]. These variables included age at diagnosis, grade, stage at diagnosis, pathologic receptor status (ER, PR, and HER2), response to neoadjuvant chemotherapy (when available), and distant metastasis-free or overall survival. TNBC was defined as being negative for ER, PR, and HER2: immunohistochemistry (IHC) 0 and FISH HER2/CEP17 ratio of less than 2.0. Neoadjuvant chemotherapy response was based on study-reported outcomes. As we previously reported, all patients in the “neoadjuvant dataset” received neoadjuvant chemotherapy but from diverse regimens: 41% of patients received anthracycline/taxane +/− alkylator, 15% anthracycline +/− alkylator, 35% taxane alone, and 9% anthracycline/platinum.

### Genomic data

For data from the METABRIC, normalized gene expression data, copy number data, and somatic mutation data for 171 cancer-related genes were obtained from the publicly available European Genome-Phenome Archive (IDs EGAD00010000210 and EGAD0001000021) and associated publications [13, 21]. Copy number segmented data files were processed using GISTIC2.0 [23]. For data from TCGA, breast cancer gene expression data, GISTIC copy number data, and somatic mutation data were obtained from the XENAbrowser (version 2015-02-24). Gene expression data from 17 published studies of breast cancer patients prior to NAC were re-processed from raw files, as previously described [7]. Genomic data from the Fudan TNBC study was downloaded from the National Omics Data Encyclopedia (accession OEP000155) [22].

### Gene expression signatures, expression-based subtypes, and inferred immune subsets

Given gene expression data from multiple studies and disparate platforms, gene expression data for all TNBCs for each dataset (METABRIC *n* = 287, TCGA *n* = 160, neoadjuvant dataset *n* = 446) were extracted, quantile normalized within TNBCs from each study, and subsequently median centered. We evaluated summary expression metrics (e.g. signatures, intrinsic subtypes, CIBERSORT proportions). One hundred twenty-five published gene expression signatures were calculated as we have previously described [7]. We determined PAM50 intrinsic breast cancer subtype using the ‘Bioclassifier’ package from Parker et al. after balancing TNBC data with an equal number of ER-positive cases for each dataset [24]. TNBC subtype was determined using the TNBCtype tool [9, 25]. Proportion of infiltrating immune cell subsets were calculated using the CIBERSORT algorithm [26].

### Modeling and performance

We compared the performance of three logistic regression models in predicting rapid relapse versus late relapse. The “null model” contained only clinical variables (age/stage at diagnosis and PAM50/TNBC subtype). The “null plus significant genomic features”, adds any feature significantly different between rrTNBC and lrTNBC with a nominal *p*-value < 0.05. The “genomic features reduced”, is a reduced version of the second model that only includes features among the top 25 most important genomic features in at least half of the independent runs. Lasso reduction and tuning of the regularization parameter lambda were performed. To evaluate model performance, we calculated the average receiver-operator characteristic (ROC) AUC of the 25 runs, and 95% confidence interval was calculated using the standard deviation of the sample of means.

### Statistical analysis

Differences in patient and tumor characteristics were evaluated using Pearson chi-squared tests. The association of gene signatures with neoadjuvant chemotherapy response was evaluated using simple linear regression and t-tests. All calculations of association were multiple-testing corrected using the Benjamini–Hochberg procedure for false discovery rate. For continuous variables, we calculated *p*-values comparing rapid vs. late and relapse vs. no relapse using ANOVA and logistic regression. For count variables (e.g. mutated vs. not) we used Fisher exact tests to evaluate relapse vs. not and rapid vs. late relapse. *P*-values for CIBERSORT and mutation signatures were evaluated using logistic regression, while CNAs, and mutations were evaluated using Fisher exact tests. Data visualization was made using ggplot2 [27]. All statistical analyses were performed in R version 3.4.1.

## Results

### Defining rapid vs. late vs. no relapse triple-negative breast cancer

**Fig. 1:**
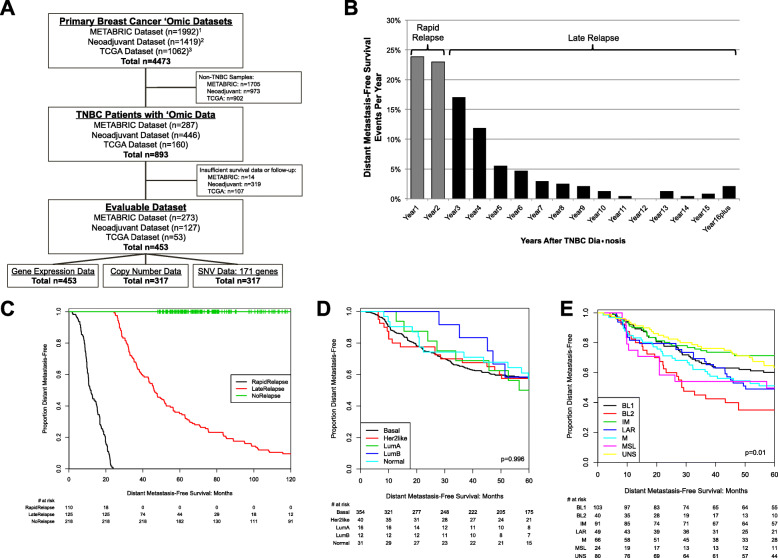
Study design and definition of triple-negative breast cancer (TNBC) rapid vs. late relapse. **a** REMARK diagram. **b** Proportion of distant metastasis-free survival (DMFS) events per year after diagnosis among evaluable dataset. ‘Rapid relapse’ was defined as DMFS events within the 2 years of diagnosis and ‘late relapse’ DMFS events beyond 2 years. **c**-**e** Kaplan-Meier diagram of DMFS in study cohort reflecting TNBC group definitions (**c**), compared with DMFS by intrinsic subtype approaches PAM50 subtype (**d**), and Lehmann TNBC subtype (**e**). *P*-value indicates log-rank test

From three large cohorts with primary breast cancer genomic data – TCGA, [12] METABRIC, [20, 21] and our prior breast cancer gene expression meta-analysis [7] – we identified 893 TNBCs from a total of 4473 breast cancer cases. For our analyses, we included patients with at least 60 months of follow-up or those with a distant metastasis-free survival (DMFS) event prior to our 60-month cutoff, leaving a total of 453 TNBCs in our evaluable dataset. Of these, 453 had gene expression data, 317 had copy number data, and 317 had mutation data. (Fig. 1a).

We assessed the percentage of total DMFS events each year (Fig. 1b). In this dataset, over 20% of DMFS events occurred each of the first 2 years after diagnosis, categorized as ‘rapid relapse’ (rrTNBC). Among lrTNBCs, most DMFS events occurred within the first 5 years after diagnosis, with sporadic events beyond year 6. Our main goal was to identify differences among TNBCs with clinically distinct outcomes, so we visualized DMFS for our relapse categorization (Fig. 1c) in comparison with DMFS for existing intrinsic expression-based subtype approaches PAM50 [24] (Fig. 1d) or Lehmann/Pietenpol TNBCtype [9] (Fig. 1e) within the same cohort. The Lehmann/Pietenpol TNBCtype (log-rank *p* = 0.01), but not PAM50, was associated with significant differences in DMFS. The strikingly different visualized outcomes suggests that our relapse categorization does, in fact, identify truly distinct subsets based on outcome when compared to approaches that focus on intrinsic features.

### Patient and tumor characteristics

We evaluated the association of clinical, pathologic, and intrinsic expression subtype with rapid vs. late vs. no relapse status (Table 1). There was no significant difference in age at diagnosis or grade, however, rrTNBCs were significantly more likely to be higher stage (Chi-square *p* = 1.9e-10). The majority of patients were basal-like PAM50 subtype (78%), but, lrTNBCs were significantly more likely to be non-basal (non-basal: rrTNBC 18%, lrTNBC 29%, nrTNBC 20%, Chi-square *p* = 0.03). Lehmann/Pietenpol TNBC subtype also reflected significant differences across groups (Chi-square *p* = 0.02). The immunomodulatory phenotype was highest in nrTNBC (16% rrTNBC, 16% lrTNBC, 24% nrTNBC), luminal androgen receptor was highest in lrTNBC (9% rrTNBC, 16% lrTNBC, 9% nrTNBC), and basal-like 2 was highest in rrTNBC (15% rrTNBC, 9% lrTNBC, 6% nrTNBC). A subset of patients in this cohort (127/453; 28.0%) had data on response to neoadjuvant chemotherapy (NAC). As anticipated, those patients with rrTNBC or lrTNBC were significantly more likely to have residual disease (RD) after neoadjuvuant chemotherapy (93 and 94% RD, respectively), relative to those with nrTNBC (51% RD; Chi-square *p* = 1.9e-7). Intriguingly, the rate of residual disease was similar among rrTNBC and lrTNBC despite markedly different timing of relapse.

**Table 1:** Cohort clinical and pathologic features

Rapid Relapse	All patients *n* = 453 n (%)	Rapid Relapse *n* = 110 n (%)	Late Relapse *n* = 125 n (%)	No Relapse *n* = 218 n (%)	*P*
Age at diagnosis, by decade					0.12
< 40 years	75 (17)	15 (14)	27 (22)	33 (15)	
40 to 50 years	117 (26)	27 (24)	28 (22)	62 (29)	
50 to 60 years	124 (27)	33 (30)	25 (20)	66 (30)	
> 60 years	137 (30)	35 (32)	45 (36)	57 (26)	
Grade at diagnosis					0.86
I	6 (2)	2 (2)	2 (2)	2 (1)	
II	54 (14)	14 (15)	17 (26)	23 (13)	
III	321 (84)	75 (83)	89 (82)	157 (86)	
Stage at diagnosis					**< 0.001**
I	73 (17)	3 (3)	18 (15)	52 (25)	
II	231 (54)	43 (44)	69 (56)	119 (58)	
III	123 (29)	52 (53)	35 (29)	36 (27)	
Pam50 Subtype					**0.03**
Basal	354 (78)	90 (82)	89 (71)	175 (80)	
Non-Basal	99 (22)	20 (18)	36 (29)	43 (20)	
TNBC Subtype					**0.02**
Basal-like 1	103 (23)	23 (21)	26 (21)	54 (25)	
Basal-like 2	40 (9)	16 (15)	11 (9)	13 (6)	
Immunomodulatory	91 (20)	18 (16)	20 (16)	53 (24)	
Luminal androgen receptor	49 (11)	10 (9)	20 (16)	19 (9)	
Mesenchymal	66 (14)	19 (17)	20 (16)	27 (12)	
Mesenchymal stem-like	24 (5)	10 (9)	3 (2)	11 (5)	
Unselected	80 (18)	14 (13)	25 (20)	41 (19)	
Response to Neoadjuvant Chemo					**< 0.001**
Pathologic complete response	29 (23)	4 (7)	1 (6)	24 (49)	
Residual disease	98 (77)	57 (93)	16 (94)	25 (51)	

### Response to Neoadjuvant chemotherapy and survival in TNBC: immune and expression signatures

**Fig. 2:**
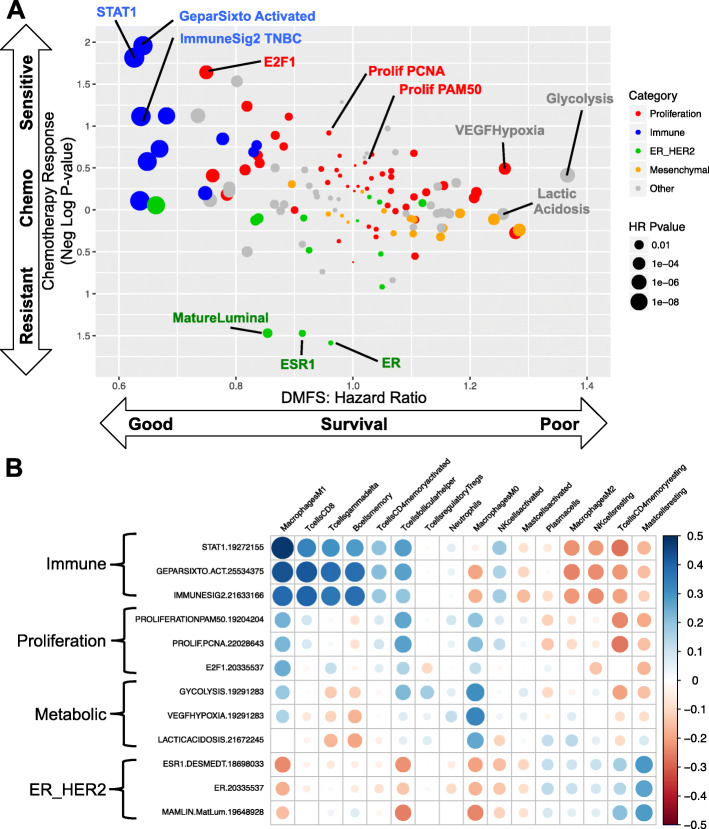
Immune and Expression Signatures and Response to Neoadjuvant Chemotherapy and Survival in TNBC. **a** The calculated score for 125 published gene expression signatures for 127 patients with data on response to neoadjuvant chemothrapy and distant metastasis-free survival (DMFS). Each signature is a point. The association of each signature with neoadjuvant chemotherapy response (pathologic complete response vs. RD) by simple linear regression (y-axis) and hazard ratio for each signature using DMFS (x-axis) are displayed. Signatures were grouped by phenotype (as previously described [7]), identified by color: proliferation signatures (red), immune signatures (blue), ER/HER2 signatures (green), mesenchymal signatures (orange), others (grey). Size of each point relates to the hazard ratio *p*-value for each signature. **b** The association of three representative signatures from each group (immune, proliferation, ER/HER2, mesenchymal) with the relative proportion of 22 inferred immune cell subsets via CIBERSORT across all samples with gene expression data (*n* = 453) are visualized using CorrPlot [26, 29].

Response to NAC is known to be a robust prognostic biomarker in TNBC [28]. In this cohort, only 28% (127/453) of patients received NAC and many of the regimens were non-standard (e.g. taxane alone). Because of this, the pathologic complete response (pCR) after NAC was only 22.8%, much lower than modern current regimens, typically ~ 40%. Despite these significant limitations, pCR was strongly associated with nrTNBC (*p* < 0.001). The patients with data on response to NAC all had whole transcriptome data but no available mutation or copy number data, so we calculated a score for 125 published gene expression signatures and evaluated the association of each signature with NAC response (pCR vs. RD) and DMFS. Signatures were grouped by phenotype as previously described [7] (*n* = 127 patients; Fig. 2a). Immune signatures were associated with better prognosis and most were also associated with improved response to NAC. Proliferation signatures tended to be associated with improved response to NAC, as we have previously described [7], yet there was variable association with DMFS.

To understand what immune cell types in the tumor microenvironment may be reflected by the immune signatures, we visualized the association of three representative signatures from each group (immune, proliferation, ER/HER2, mesenchymal) with the relative proportion of 22 inferred immune cell subsets via CIBERSORT (Fig. 2b) [26]. Immune signatures were strongly positively correlated with anti-tumor immune cell types including M1 macrophages, CD8 T-cells, and memory B-cells (all Pearson’s r ≥ 0.3, all *p* < 1.2e-8) and anti-correlated with immune suppressive cell types including M2 macrophages, memory resting CD4 T-cells, resting NK cells, and resting mast cells. ER/HER2 signatures reflected an almost opposite pattern to immune signatures, with positive correlation to immune suppressive cell types and anti-correlation with anti-tumor immune cell type. Metabolic signatures appeared to have a strong correlation specifically with M0 macrophages (all Pearson’s *r* > 0.27, all *p* < 8.4e-9). As a sensitivity analysis, we evaluated the association of three representative signatures from each group with 7 immune cell-type specific signatures from MSigDB [30, 31] (instead of CIBERSORT) and found similar results (Supplementary Figure 1A).

### Expression signatures in rapid vs. late vs. no relapse TNBC

**Fig. 3:**
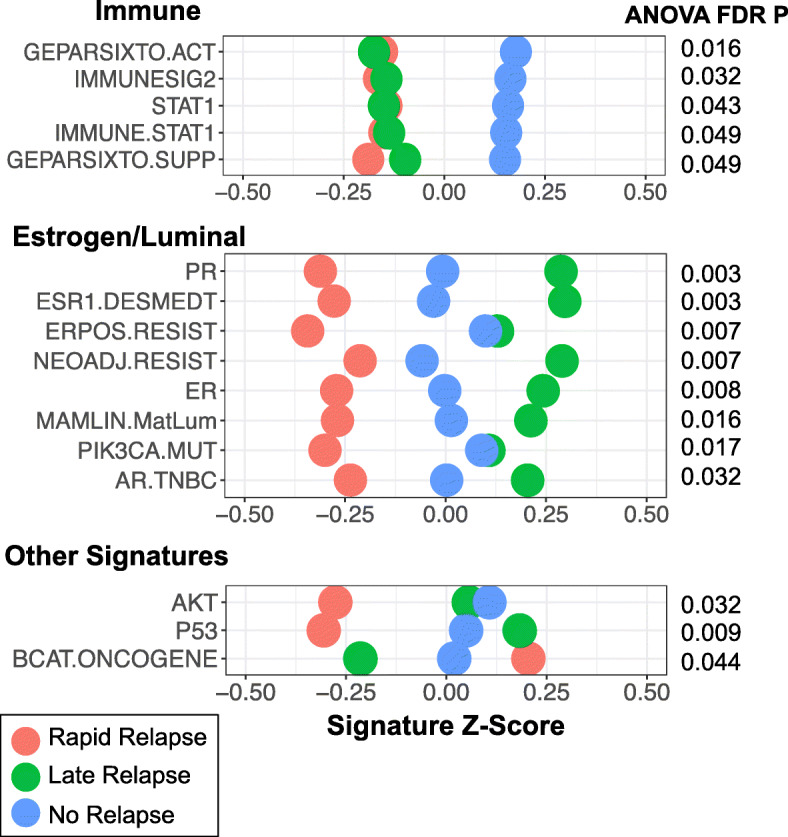
Expression Signatures in Rapid vs. Late vs. No Relapse TNBC. The calculated score for 16 published gene expression signatures that demonstrated statistical significance (ANOVA FDR *p* < 0.05) comparing rapid vs. late vs. no relapse. Signatures visualized as relative values (Z-score) with rapid relapse (red), late relapse (green), and no relapse (blue)

To assess pathways and phenotypes associated with rapid vs. late vs. no relapse, a score was calculated for 125 published gene expression signatures across the entire dataset (Supplementary Figure 1B). Evaluating each signature individually across the three groups revealed 16 signatures that were significantly different (ANOVA FDR *p* < 0.05; Fig. 3, Supplementary Figure 2A-B). Among these, five signatures were immune-related [9, 32–34] and all were significantly higher in nrTNBC relative to rrTNBC and lrTNBC. Eight significant signatures were related to luminal phenotype – all were highest in lrTNBC, lowest in rrTNBC, and intermediate in nrTNBC. While we and others have demonstrated that proliferation signatures are strongly associated with response to neoadjuvant chemotherapy independent of immunophenotype [7, 35] as well as overall survival, [36] we did not identify a significant association of proliferation signatures across all three groups (Supplementary Figure 2B). However, when evaluating rapid versus late relapse only as an exploratory analysis, late relapse was associated with significantly lower proliferation, for example the PAM50 proliferation score (t-test *p* = 0.007). Most CIBERSORT immune subsets were not statistically significant (Supplementary Figure 2C), however, neutrophils were significantly higher in rrTNBC (ANOVA FDR *p* = 0.001). To more comprehensively investigate inferred immune subsets, we evaluated the association of summed protumorigenic subsets (Tcells-CD4 naive, Bcells-naive, Mast cells-resting, NK cells-resting, Tcells-CD4 memory resting, Plasma cells, Dendritic cells resting, Tcells-regulatory/Tregs, Macrophages-M0, MacrophagesM2) and summed antitumorigenic subsets (Monocytes, Eosinophils, Tcells-gamma delta, Tcells-follicular helper, Tcells-CD8, NK cells-activated, Bcells-memory, Mast cells-activated, Neutrophils, Macrophages-M1, Dendritic cells-activated, Tcells-CD4 memory activated) with rapid versus late versus no relapse (Supplementary Figure 2D). Antitumorigenic subsets were significantly different among relapse groups (ANOVA *p* = 0.002), highest in ‘no relapse’, while there was no difference in the protumorigenic subsets (ANOVA *p* = 0.62), although the absolute differences were small.

### Mutations and copy number alterations

**Fig. 4:**
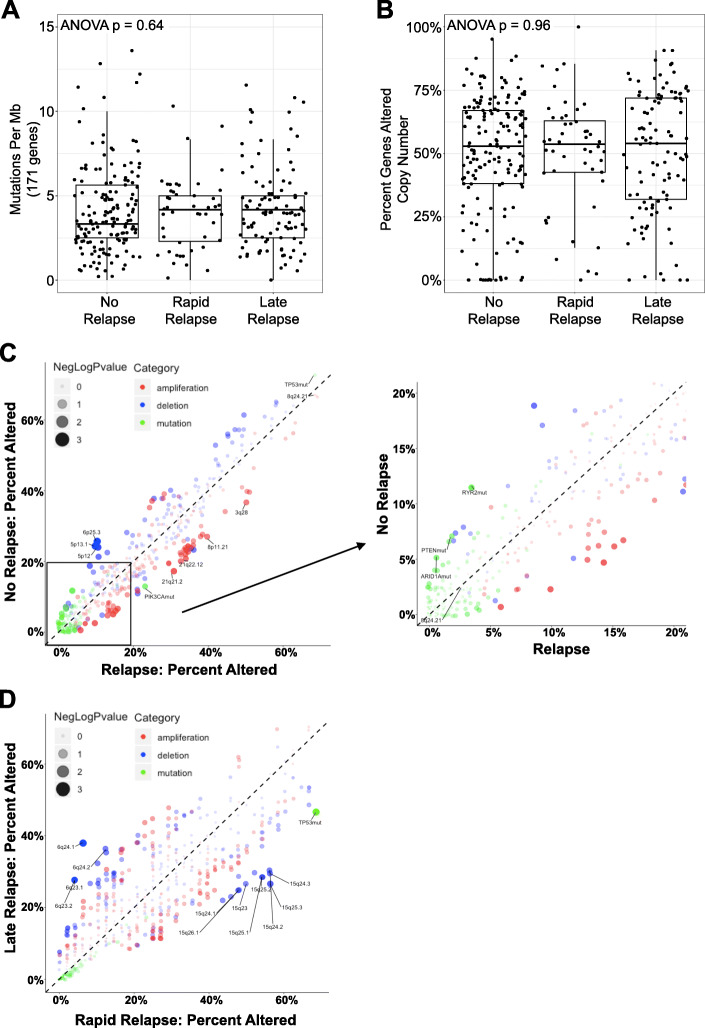
Mutations and copy number alterations in rapid vs. late vs. no relapse TNBCs. **a** Mutations per megabase of 171 cancer-related genes. **b** Percent genes altered by copy number gain (GISTIC 1 or 2) or loss (GISTIC −1 or − 2). **c** Frequency of alteration of 171 cancer-related genes (green dots), copy number gains (red dots) or losses (red dots) by cytoband among rapid relapse (x-axis) vs. no relapse (y-axis) TNBCs (**c**) or rapid relapse (x-axis) vs. late relapse (y-axis) TNBCS (**d**). Size of dot indicates negative log of *p*-value for Fisher exact test with those genes and cytobands indicated demonstrate nominal *p* < 0.05. Zoomed-in image of those alterations with < 20% frequency indicated in right panel

In this cohort, 70% (317/453) of patients had data on single nucleotide variant/mutation data including 171 cancer-related genes and whole genome CNAs [21]. Only a small subset of patients (11.7%; 53/453) had whole exome mutation data, so we focused on the 171 cancer-related genes to ensure adequate statistical power. When evaluating general mutational features, there was no significant difference in mutations per megabase (ANOVA *p* = 0.64; Fig. 4a) nor percent genome altered by copy number (ANOVA *p* = 0.96; Fig. 4b).

We first compared the frequency of alteration for each mutation and cytoband (for CNAs) for relapse (rrTNBC + lrTNBC) vs. nrTNBC (Fig. 4c) because of low mutation frequency for most genes. There were no genes that were significantly different after multiple testing (Supplementary Figure 3A) when comparing relapse vs. no relapse, but *PIK3CA* mutations were more frequent in relapse relative to nrTNBC. In addition, *PTEN, ARID1A,* and *RYR2* mutations were enriched in nrTNBC relative to rrTNBC (Fisher exact nominal *p* < 0.05). We then compared rrTNBC vs. lrTNBC (Fig. 4d) and found that rrTNBC were significantly more likely to harbor a mutation in *TP53* compared to lrTNBC patients (Fisher exact FDR *p* = 0.009). Among CNAs, the copy number landscape was similar across the rapid vs. late vs. no relapse groups (Supplementary Figure 3B) and there were no significantly altered genes or regions among these three groups after multiple test correction yet there were several regions that demonstrated enrichment within specific groups (nominal *p* < 0.05; Fig. 4c-d).

### Clinical and multi-‘omic model of rapid vs. late relapse in TNBC

**Fig. 5:**
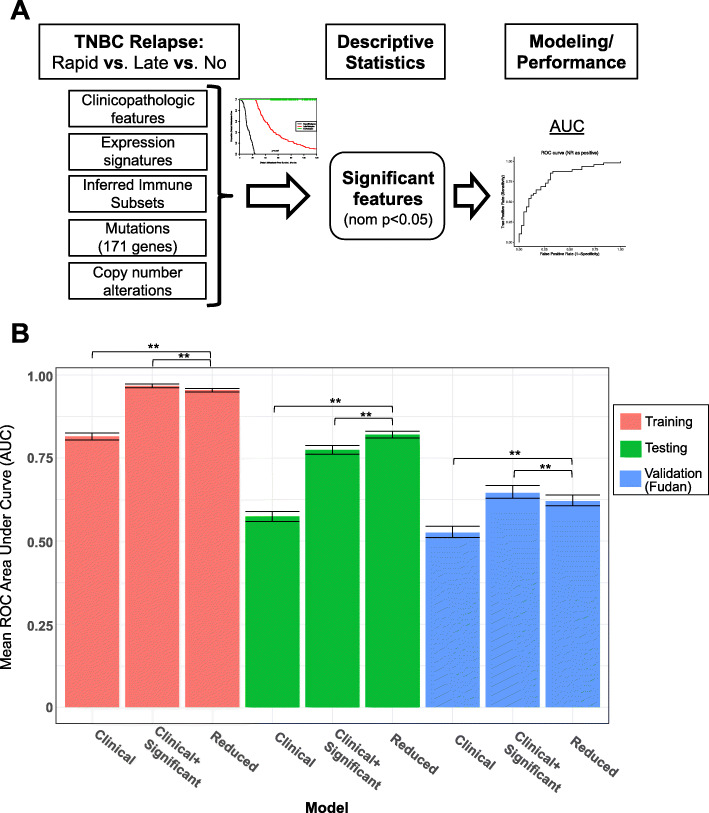
Developing an optimal clinical and multi-‘omic model of rapid vs. late relapse in TNBC**. a** Schematic of experimental steps including definition of variables, descriptive statistics, comparative modeling including model tuning, and assessment of model performance. **b** Receiver-operator characteristic (ROC) plots for each model’s performance, measured by average area under the curve (AUC) of 25 independent runs of the train-test split. Each model was tuned to ensure optimal performance. Models are grouped and colored by cohort—red indicates training data (*n* = 63), green indicates testing data (*n* = 63), and blue indicates the independent validation Fudan cohort (*n* = 34). For each grouping, the three models shown are: 1) “null model”, including only clinical variables; 2) “null plus significant features”, adding any feature significantly different between rrTNBC and lrTNBC with a nominal *p*-value < 0.05; and 3) “null plus significant features reduced”, including only features from model 2 that are among the top 25 most important genes in at least half of the independent runs. Asterisks indicate significance by Wilcoxon rank sum, * indicates *p* < 0.05, ** indicates *p* < 0.01, NS indicates “not significant” (*p* > 0.05)

Having identified discrete clinical, expression, immune, mutation, and copy number features among primary TNBCs with distinct clinical outcomes, we sought to develop an optimal, multi-‘omic predictive model for rrTNBC vs. lrTNBC. We compared performance of three logistic regression models with lasso reduction (detailed in the Methods; Fig. 5a). The clinical, “null model”, performed marginally in both the testing cohort and the independent validation cohort (average AUC 0.574 and 0.525, respectively). The other two models (clinical+genomic and reduced genomic) had significantly improved performance in both the testing cohort and the independent validation (average AUC: 0.774 and 0.821 for testing; 0.645 and 0.620 for validation; Fig. 5b; all Wilcoxon rank sum *p* < 0.005). The genomic features that contributed most included clinical features (stage, expression subtypes), mutations (*ARID2*, *DNAH11*, *SETDB1*), copy number alterations (loss *LAMA2, CLK3, MLLT4, SYNE1* and gain *DNAH5, LIFR, PETN*), and expression signatures (signatures of RBBP8 [37], ER negative chemoresistance [38], PTEN deletion [39], beta catenin [40], STAT3 [41], and RAS pathway activation [42]). We evaluated additional models, including machine learning approaches (random forest, support vector machine) as well as the universe of available genomic data, however, these additional modeling approaches were characterized by overfitting even in the context of model tuning and demonstrated no significant improvement in performance relative (data not shown).

## Discussion

We previously demonstrated in two large cohorts that disparities in sociodemographic features are strongly associated with rrTNBC, [17–19] and in this report sought to investigate genomic features associated with rrTNBC. We aggregated data from the available cohorts that have multi-‘omic data as well as adequate follow-up to characterize TNBCs as rrTNBC, lrTNBC, or nrTNBC. Although limited by the retrospective nature and limited number of patients who received modern NAC, we provide initial observations regarding genomic features and rrTNBC.

Our goal was to identify distinguishing features and determine if predictive models incorporating clinical, expression-based subtype, and/or multi-‘omic models could identify patients at high risk of rapid relapse. lrTNBCs are more likely to be non-basal (primarily luminal A/B) and our data identify eight luminal signatures are associated with late relapse. Multiple groups have identified a ‘luminal androgen receptor’ subset of TNBC based on molecular classifications, [9, 11] and 40% (20/49) of the Lehmann LAR subtype tumors in our cohort ultimately had late relapse. To develop predictive models, we first identified the relatively few specific features that were significantly different across subsets (61 features from > 35,000 initial data points) then built models based on a priori feature identification. This approach led to overall good performance of multiple models, and importantly allows us to understand what genomic features contribute most. More complex modeling approaches (e.g. machine learning algorithms) did not improve model performance and led to challenges with overfitting. Our models were evaluated in over two times the number of TNBC patients available in TCGA [12] alone – a remarkable number for a disease that accounts for only approximately 15% of breast cancers [1, 2]. Collectively, our data support the categorization by Burstein et al. [11] and suggest that lrTNBCs are enriched for luminal phenotypes while rrTNBCs are likely enriched for the ‘basal-like immune suppressed’ phenotype.

Stage at diagnosis was strongly associated with rrTNBC in univariate analyses and in logistic regression models. One hypothesis is that stage at diagnosis captures non-biological features including socioeconomic or demographics features [43–45]. Race/ethnicity is complex, [46, 47] was largely unavailable in the included datasets, and warrants further study [48, 49]. In a parallel study, we investigated the association of sociodemographic features with rrTNBC among 3016 primary TNBCs at ten academic cancer centers [50]. In this large cohort, we found that stage at diagnosis remained significant, as well as Medicaid/indigent insurance, lower income, and younger age [50]. Collectively, this suggests that timing of relapse is impacted by a complex set of clinical, genomic, and sociodemographic features that warrant further multi-level analyses.

Response to neoadjuvant chemotherapy remains the best prognostic biomarker for TNBC, [28] but there are clear differences in disease course among TNBCs who develop relapse earlier vs. later. At the time of these analyses, no large multi-‘omic dataset including NAC and long-term outcomes were available although this is anticipated in the future. Despite significant limitations of NAC analyses, somewhat unexpectedly patients destined for rrTNBC and lrTNBC in this cohort had similarly high rates of residual disease to neoadjuvant chemotherapy. Both rrTNBC and lrTNBC had lower expression of immune signatures compared with nrTNBCs, reflecting reduced anti-tumor immune response. This supports our and others’ work, [7, 51–54] including our analyses of the BrighTNess phase III clinical trial, which provides largest transcriptome dataset and association with NAC and demonstrated that stratifying patients by proliferation and immune signatures can effectively stratify likelihood of pCR irrespective of NAC regimen. Given the recent FDA approval of immunotherapy for metastatic TNBC [6], there is great interest to augment the existing host anti-tumor immune response [55–58].

Clinically, it is clear that a subset of patients with TNBC have highly aggressive, largely treatment-refractory disease [1, 2, 14]. In the modern era, NAC offers a biological ‘readout’ of chemosensitivity that is highly associated with both recurrence and survival endpoints and has become standard of care, with pathologic response used to guide subsequent escalation/de-escalation of adjuvant therapy [59]. However, among the highest risk TNBCs with RD after NAC, we still have limited ability to identify the ~ 40% patients destined for relapse [7, 8]. We envision that the results of this and similar efforts, such as circulating tumor DNA minimal residual disease assays, [60–62] could identify patients at highest risk (rrTNBCs in the current study) and direct these patients to escalation of therapy, additional maintenance therapy, and/or intensive monitoring.

While this study presents promising methods to categorize TNBC relapse it does possess significant limitations. Categorization of tumors depends on study-reported estrogen receptor (ER) status; variability and changes in standard determination of estrogen receptor positivity since 2010 guidelines [63] may have influenced whether a subset of tumors included had very low ER (e.g. < 10%). The lack of available robust multi-‘omic datasets with long-term outcome data leads to inherent limitations of aggregating multiple datasets. We incorporated genomic data from multiple studies, generated using multiple platforms, and over multiple years. While we have attempted to account for this through standard normalization approaches and analysis only of summary statistics (e.g. expression signatures not individual genes), batch/platform effects and computational analyses could impact our results. For assessment of tumor mutation burden, we used mutation data from a 317 gene targeted panel assay. While several studies suggest that TMB by targeted panel overall correlates with whole exome or whole genome sequencing, these methodologies are not identical [64–66]. Therapy for TNBC has changed, including: 1) standard use of neoadjuvant chemotherapy for nearly all patients with TNBC, while not all patients included received neoadjuvant or adjuvant therapy, particularly in METABRIC (161/273; 59.0%); 2) incorporation of capecitabine for RD based on CREATE-X [67]; and 3) recent FDA approval of immunotherapy for metastatic, PD-L1 positive TNBC [6].

In conclusion, we provide evidence that rrTNBC reflects a distinct clinical entity characterized by unique genomic features. Predictive modeling using clinical and genomic features in these datasets revealed modest results, but with improved data may identify patients at high risk for ‘rapid relapse.’ Multi-level analyses of the interaction between clinical, multi-‘omic, and sociodemographic features and timing of relapse are warranted.

## Supplementary Information

**Additional file 1: Figure S1**. Additional Analyses of Gene Expression Signatures. **(A)** Sensitivity analyses of correlation between three representative signatures from each group (immune, proliferation, ER/HER2, mesenchymal) with the immune cell-specific signatures [30, 31] across all samples with gene expression data (*n* = 453), visualized using CorrPlot [26, 29]. **(B)** Heatmap with hierarchical clustering of the gene expression signatures with the greatest variance (top 25%) across the dataset. **Figure S2**. Variation of Expression Signatures Across Rapid vs. Late vs. No Relapse Groups. The calculated score for 16 published gene expression signatures that demonstrated statistical significance (ANOVA FDR *p* < 0.05) comparing rapid vs. late vs. no relapse. The score value is presented for immune signatures **(A)** and estrogen/luminal signatures **(B)**. Each boxplot represents the 25th to 75th percentile with the median indicated as the central line and whiskers indicating 1.5 x interquartile range. **(C)** Immune cell subset proportion from CIBERSORT, visualized as relative values (Z-score) with rapid relapse (red), late relapse (green), and no relapse (blue). **Figure S3**. Mutation and Modeling Sensitivity Analyses. **(A)** CoMut plot of gene-level mutation for the entire cohort, with mutation indicated in blue, visualized with ‘GenVisR’ package [68]. **(B)** Frequency of gene-level copy number gains (red) or losses (blue) across the genome

## Data Availability

Data that support the findings of this study have been deposited in the following repositories. Neoadjuvant dataset: Raw gene expression data and paired clinical feature data were obtained from NCBI Gene Expression Omnibus (GEO) via accession numbers GSE8465, GSE16446, GSE18728, GSE19697, GSE20194, GSE20271, GSE21974, GSE21997, GSE22093, GSE22226, GSE22358, GSE22513, GSE23988, GSE25066, GSE28796, and GSE32646). METABRIC: Molecular Taxonomy of Breast Cancer International Consortium. Normalized gene expression data, copy number data, and paired clinical feature data were obtained from the publicly available European Genome-phenome Archive (IDs EGAD00010000210 and EGAD0001000021) with mutation data from Pereira, et al. [21]. TCGA: Normalized gene expression data, copy number data, single nucleotide variant, and paired clinical feature data TCGA data (2015-02-24 datafreeze) were obtained from the University of California, Santa Cruz cancer browser (https://xenabrowser.net/heatmap/). Fudan dataset: Data can be viewed in The National Omics Data Encyclopedia (NODE) (http://www.biosino.org/node), accession OEP000155. Microarray data and sequence data are available in NCBI GO (OncoScan array; GEO: GSE118527) and Sequence Read Archive (WES and RNA-seq; SRA: SRP157974).

## Abbreviations

TNBC: Triple-negative breast cancer
rrTNBC: Relapse/death ≤2 years of diagnosis
lrTNBC: Relapse/death > 2 years
nrTNBC: >5 years no relapse/death.
METABRIC: Molecular Taxonomy of Breast Cancer International Consortium
TCGA: The Cancer Genome Atlas
ER: Estrogen receptor
PR: Progesterone receptor
IHC: Immunohistochemistry
ROC: Receiver-operator characteristic
