# Supplementary figures and images for: Genomic features of rapid versus late relapse in triple negative breast cancer
Source: BMC Cancer. 2021 May 18;21:568. doi: 10.1186/s12885-021-08320-7 (PMC8130400; doi:10.1186/s12885-021-08320-7)

# Supplementary Figure 1

A

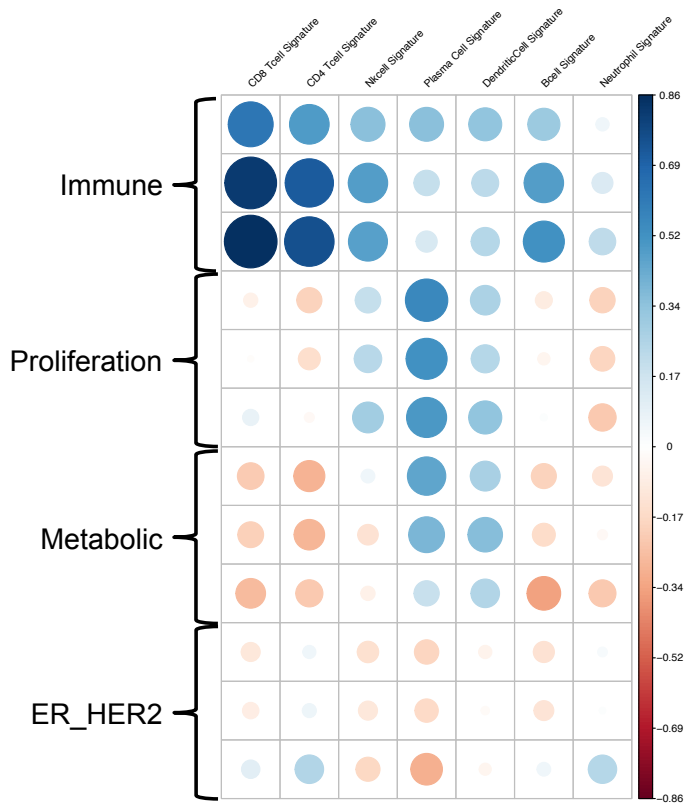

B

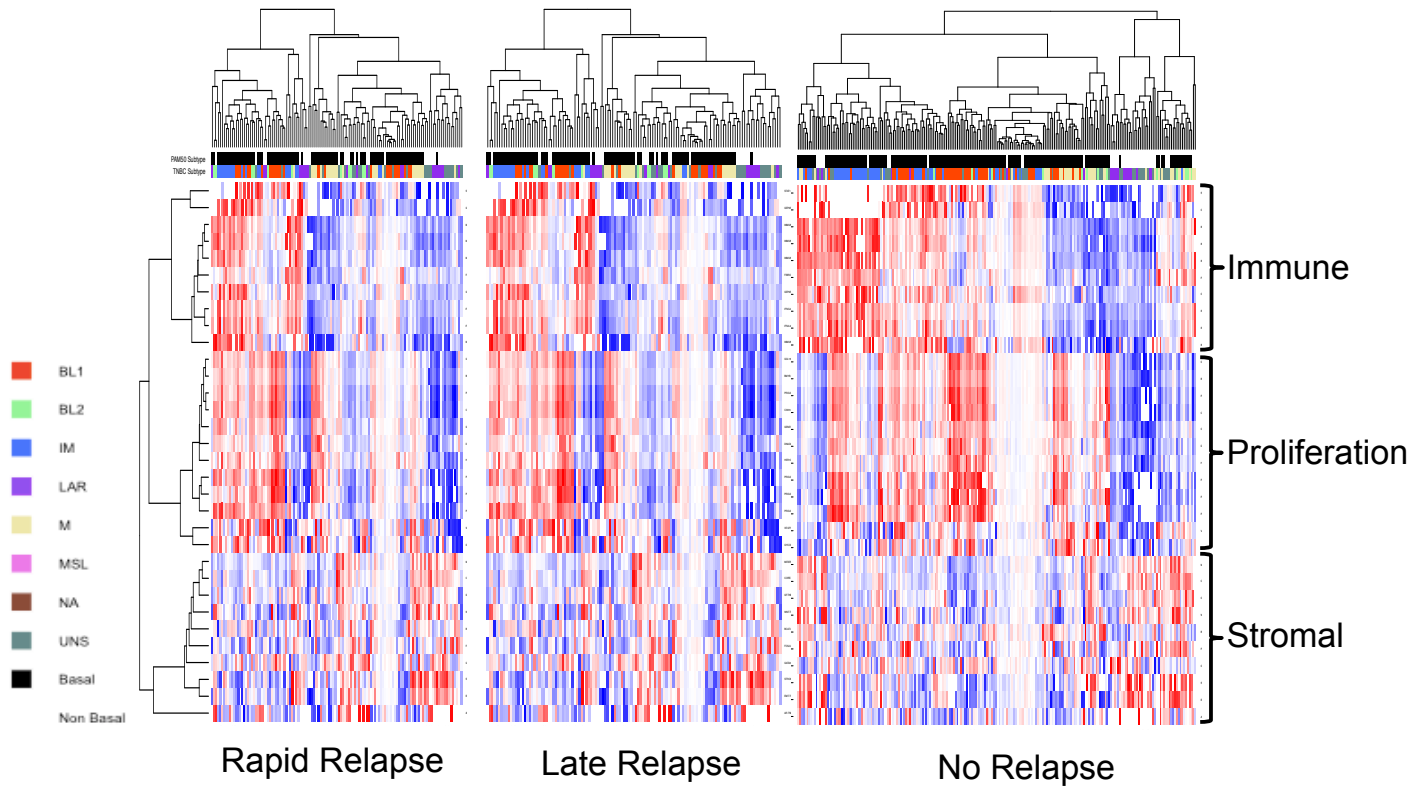

Supplementary Figure 2

A

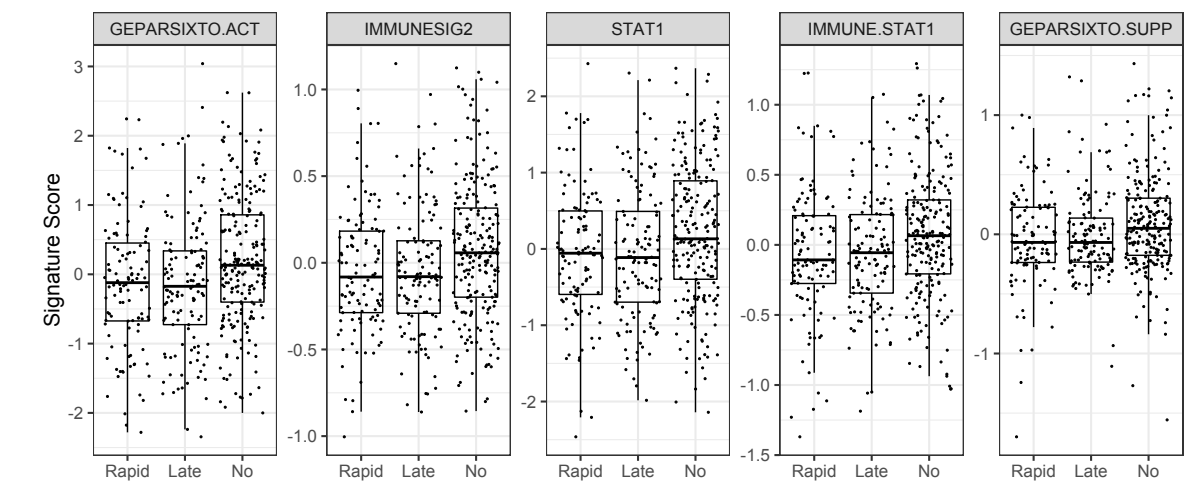

B

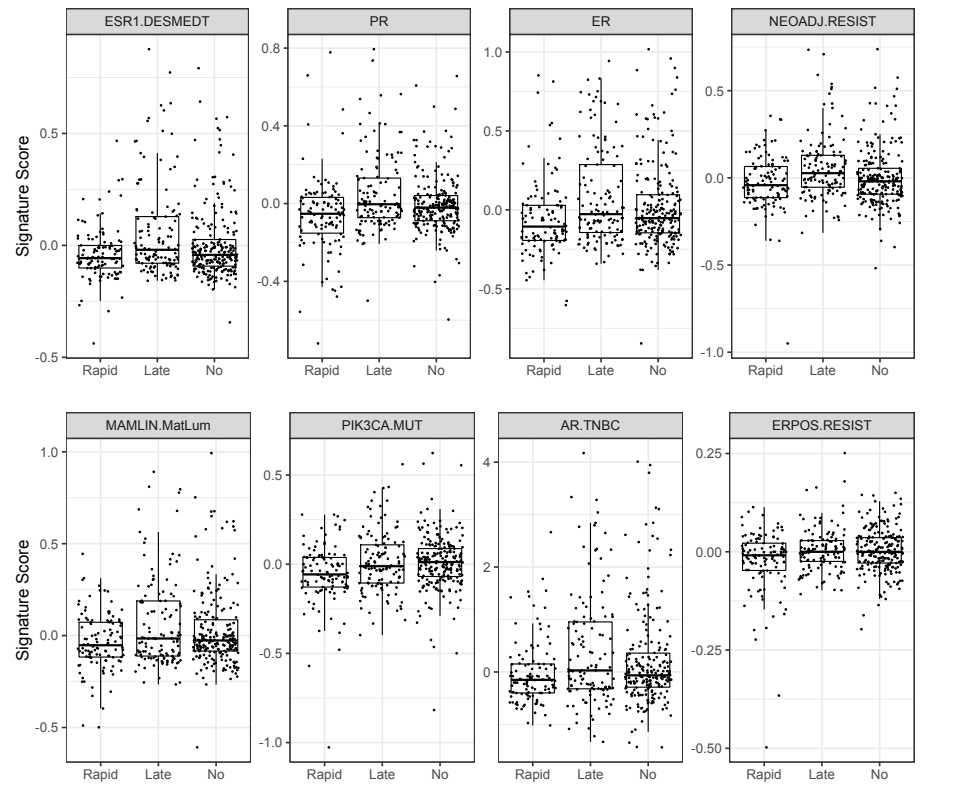

C

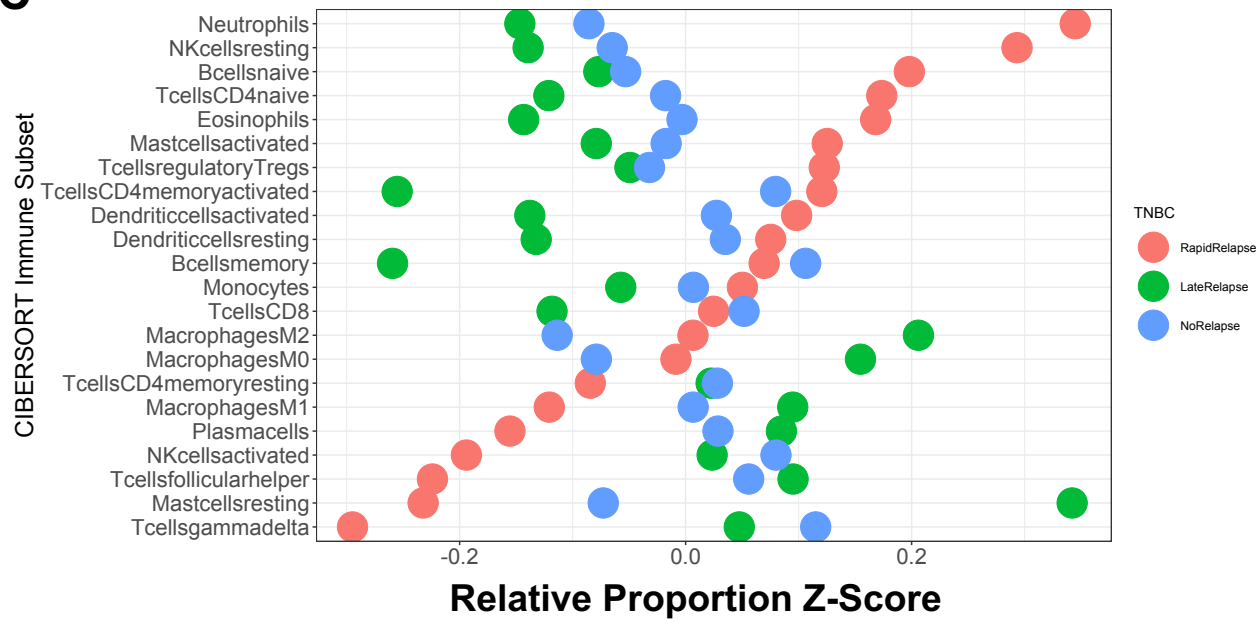

D

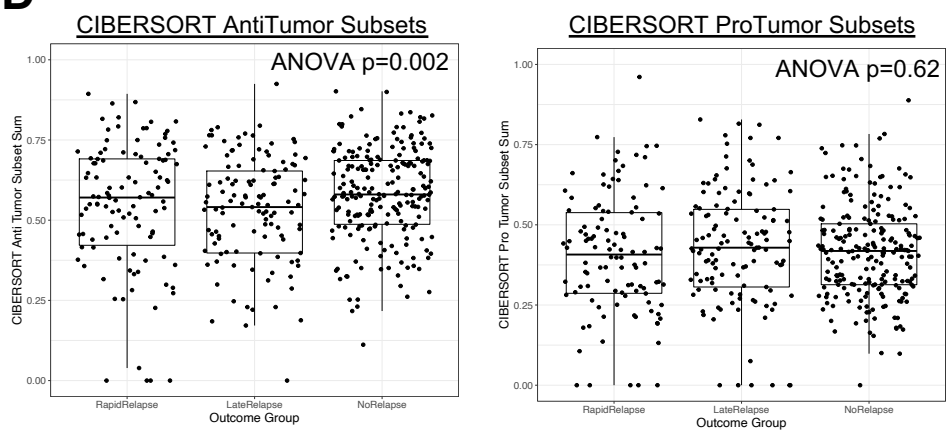

# Supplementary Figure 3

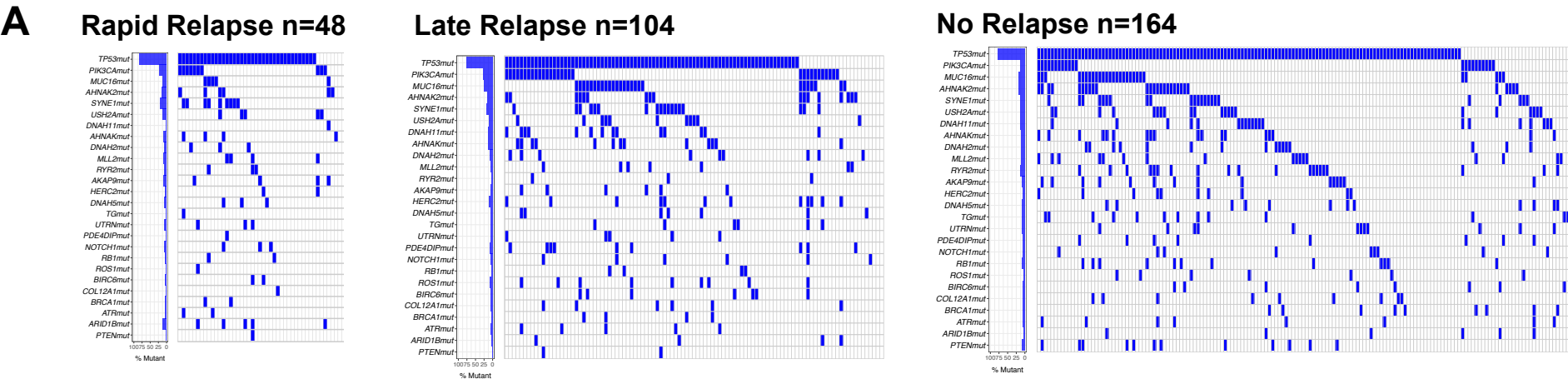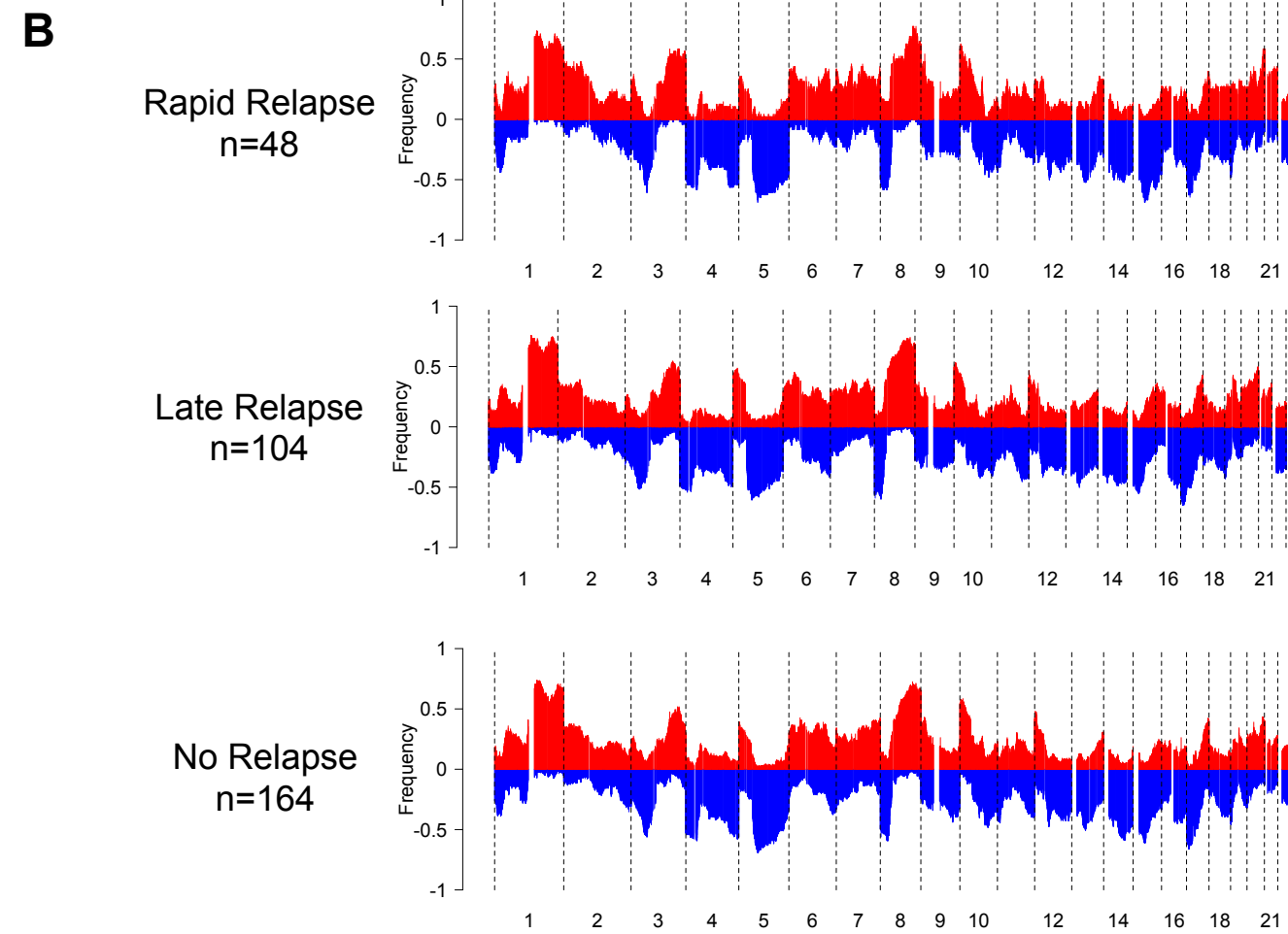

Supplement: Supplementary file 1 — Additional file 1: Figure S1. Additional Analyses of Gene Expression Signatures. (A) Sensitivity analyses of correlation between three representative signatures from each group (immune, proliferation, ER/HER2, mesenchymal) with the immune cell-specific signatures [30, 31] across all samples with gene expression data (n = 453), visualized using CorrPlot [26, 29]. (B) Heatmap with hierarchical clustering of the gene expression signatures with the greatest variance (top 25%) across the dataset. Figure S2. Variation of Expression Signatures Across Rapid vs. Late vs. No Relapse Groups. The calculated score for 16 published gene expression signatures that demonstrated statistical significance (ANOVA FDR p < 0.05) comparing rapid vs. late vs. no relapse. The score value is presented for immune signatures (A) and estrogen/luminal signatures (B). Each boxplot represents the 25th to 75th percentile with the median indicated as the central line and whiskers indicating 1.5 x interquartile range. (C) Immune cell subset proportion from CIBERSORT, visualized as relative values (Z-score) with rapid relapse (red), late relapse (green), and no relapse (blue). Figure S3. Mutation and Modeling Sensitivity Analyses. (A) CoMut plot of gene-level mutation for the entire cohort, with mutation indicated in blue, visualized with ‘GenVisR’ package [68]. (B) Frequency of gene-level copy number gains (red) or losses (blue) across the genome [file 12885_2021_8320_MOESM1_ESM.pdf]
